# What Happens When People with Depression Gather Online?

**DOI:** 10.3390/ijerph18168762

**Published:** 2021-08-19

**Authors:** Xuening Wang, Xianyun Tian, Xuwei Pan, Dongxu Wei, Qi Qi

**Affiliations:** School of Economics and Management, Zhejiang Sci-Tech University, Hangzhou 310018, China; xueningwang_zstu@outlook.com (X.W.); panxw@zstu.edu.cn (X.P.); dongxuwei_zstu@outlook.com (D.W.); qiqi_zstu@outlook.com (Q.Q.)

**Keywords:** social media, online community, depression, mental health, Sina Weibo, super topic

## Abstract

Depression is a common mental disease that impacts people of all ages and backgrounds. To meet needs that cannot otherwise be met, people with depression or who tend to suffer from depression often gather in online depression communities. However, since joining a depression community exposes members to the depression of others, the impact of such communities is not entirely clear. This study therefore explored what happens when people with depression gather in Sina Weibo’s Depression Super Topic online community. Through website crawling, postings from Depression Super Topic were compared with postings from members’ regular timelines with respect to themes, emotions disclosed, activity patterns, and the number of likes and comments. Topics of distilled postings covering support, regulations, emotions and life sharing, and initiating discussions were then coded. From comparison analysis, it was found that postings in the Depression Super Topic community received more comments and disclosed more emotions than regular timelines and that members were more active in the community at night. This study offers a picture of what occurs when people with depression gather online, which helps better understand their issues and therefore provide more targeted support.

## 1. Introduction

Depression is one of the most common mental disorders, typically manifested by persistent sadness, hopelessness, and pessimism [1,2]. According to the World Health Organization, more than 322 million people struggle with depression [1], and the number showed an upward trend of 49.86% between 1990 and 2017 [3]. Various approaches have been applied to help people with depression. The approaches include medication, neuro-feedback techniques, psychological counseling, and support from laypersons [4,5,6,7]. Despite the practical help available to fight depression, few people seek help actively due to the general population’s misunderstanding of depression. For example, people may hold the belief that people with depression are dangerous, weak, and self-centered [8,9]. Against the backdrop of Chinese culture, depression is often considered an excuse for an individual’s failure and can bring shame to their family [10,11]. Rather than receiving support and help, people with depression often face discrimination in the workspace, at schools, and among family and friends [10,12]. While facing a lack of support in the real world, social media may be a platform for seeking support online.

The anonymity of social media enables people with depression to socialize, get information, and vent emotions freely [13,14,15], which makes social media platforms a potentially effective tool for studying depression-related issues. By exploring the patterns of users’ online behaviors on social media, depression can be identified and predicted. Specifically, Nadeem proposed a “bag of words” method to recognize depression and found that his model achieved an F1-Score (a measure of a model’s performance) as high as 0.86 [16]. Based on deep learning, Wang jointly considered users’ postings and posting behavior to predict depression and achieved an F1-Score of 0.9772 [17]. Unlike traditional methods, such as interviews and scales [18,19], studying online platform-using patients with depression could help us understand the whole group on a large sample but at a low cost.

Other researchers have focused on online communities formed by people with depression. For example, Feldhege summarized different participation styles of members in an online depression community [20]; Park found that interactions in the online community could significantly improve individuals’ psychological state [21]; and Tang found differences in emotions, informational support, and social support between communities with management approaches and those without [22]. Despite the foregoing research, there are many questions that remain to be answered. For example, whether people with depression are more willing to express themselves in an online community; whether the online community will influence their diurnal activity pattern; and whether the online community is seen as a place to vent negative emotions, to seek peer support, to share positive emotions, or deliver support. The answers to those questions may help to provide targeted help for people with depression.

With more than 500 million monthly active users, Sina Weibo is the largest social medium in China. As the Chinese equivalent of Twitter, people post messages and images on Sina Weibo. The postings people upload on Sina Weibo are displayed in reverse chronological order on their profile pages and followers’ home pages, so a list of a user’s postings is also called a regular timeline. Super Topic is an online community in Sina Weibo that attracts users with the same interests to share information and express their feelings. In Super Topic, every user can apply to establish a sub-community or be a moderator in an existing sub-community. Depression Super Topic is one of the sub-communities established for people with depression, mainly formed by those suffering from depression along with a small number of careers. In Depression Super Topic, members can post messages and interact with others through liking, commenting, and reposting.

To help investigate what happens when people with depression gather online, this study posed the following research questions (RQ):

RQ1. How are online depression communities self-administered and self-guided?
RQ2. What are the differences in the postings’ content between Depression Super Topic and members’ regular timelines?
RQ3. What are the differences in interactions between individuals in Depression Super Topic and their regular timelines?
RQ4. What are the differences in activity patterns between individuals in Depression Super Topic and their regular timelines?

## 2. Materials and Methods

Data used in this study are publicly available. Since we did not invade any individuals’ privacy by disclosing users’ identities, there were no ethical issue to address.

### 2.1. Data Collection

Among all the sub-communities dealing with Depression Super Topic, Depression Super Topic is the largest, with more than 697,000 posts and more than 280,000 members up to April 2021. Between 4 to 9 April, 203,659 posts written by 39,071 members from this online community were crawled, and those postings were uploaded from 3 September 2017 to 9 April 2021. Apart from the text of the postings, other features were also obtained, such as posters’ user IDs, posting time, number of comments, and number of likes.

**Figure 1:**
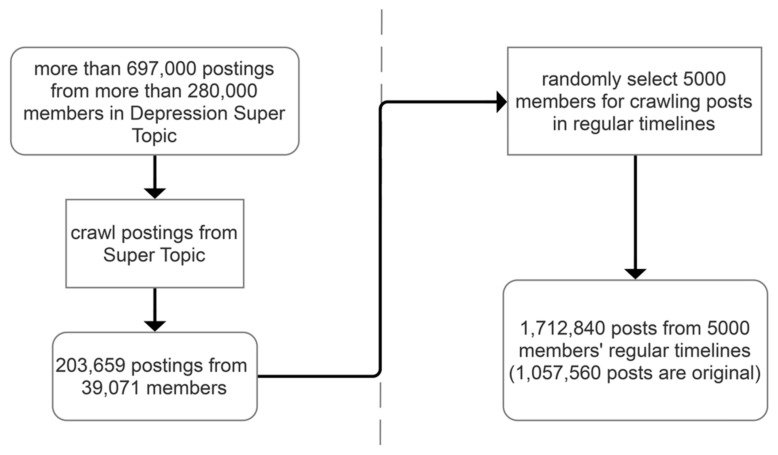
Flowchart of data collection from members of Depression Super Topic.

To explore the difference between users’ participating in regular timelines and Depression Super Topic, we collected the users’ activity data in these two different places. However, since the number of postings in the 39,071 members’ regular timelines was too large, the timelines of 5000 were randomly selected for crawling. Finally, 1,712,840 posts from regular timelines were obtained, from which 1,057,560 were original postings and 655,280 were reposts. Figure 1 provides a flowchart of data collection.

### 2.2. Themes of Distilled Posts

In Depression Super Topic, the community is administrated by moderators, who are also community members. Moderators select posts of high quality and popularity as distilled posts that they wish members to pay attention to. Distilled posts reflect this community’s focus and how it is self-guided. A total of 239 distilled posts were crawled.

Two researchers read the 239 distilled posts and established an initial coding book. The coding book includes five themes: (1) emotional support; (2) informational support; (3) regulations on community management and warnings of undesirable content; (4) emotional disclosure and life sharing; and (5) initiating discussions. Two researchers independently coded the posts based on the coding book, and disagreements were discussed to reach a consensus on the final assigned code. To check the inter-coder reliability, a third member was trained to code the total distilled posts. Inter-coder reliability for each theme was as follows: (1) emotional support: percent agreement 87.7%, kappa 0.88; (2) informational support: percent agreement 94.5%, kappa 0.92; (3) regulations on community management and warnings of undesirable content: percent agreement 96.55%, kappa 0.97; (4) emotional disclosure and life sharing: percent agreement 96.36%, kappa 0.91; and (5) initiating discussions: percent agreement 92.86%, kappa 0.92.

### 2.3. Comparison Analysis of Topics, Sentiment, Interaction, and Diurnal Activity Pattern

Because reposts may contain some noisy information, which may influence the analysis of topics, sentiment, and interaction, reposts were removed for those analyses. Considering reposting and posting are both activities in Sina Weibo, a total of 1,712,840 postings were used to explore members’ diurnal activity patterns.

The Latent Dirichlet Allocation (LDA) model was employed to investigate the difference of topics between regular timelines and Depression Super Topic [23]. When using an LDA model, the number of topics (K) must be given by the researcher. To choose the best number of topics, our research team built models with different values of K and measured them based on C_V_ coherence [24]. Initially, we built two groups of models with K = 10, 20, 30, 40, 50, 60, 70, 80, 90, and 100. One group was based on 200,000 original postings randomly selected from users’ regular timelines, and the other was based on Depression Super Topic. The model with K = 10 showed the highest value for both groups, so LDA models with K between 2 and 19 (except model with K = 10 that has been built) were constructed in the second run to compare the C_V_ coherence value. For models based on texts from Depression Super Topic, C_V_ coherence arrived at the highest point when K = 6. For the other group of models, K = 4 had the highest C_V_ coherence. We therefore chose 6 and 4 as the value of K for Depression Super Topic and regular timelines. LDA models return keywords for each topic, so we labeled the topics by observing the postings and the top 30 characteristic keywords. Based on manual judgement, two topics in regular timelines were all about celebrities, so those two were integrated into one. Finally, there were three topics found in regular timelines and six in Depression Super Topic.

In order to understand what emotions individuals vented in depression communities online and whether there are differences between emotions vented in the depression community and regular timelines, a Simplified Chinese Linguistic Inquiry and Word Count (SCLIWC) dictionary was used to conduct an emotion analysis [25]. SCLIWC analyzed a text from 102 dimensions and returned a score for each dimension, representing the proportion of the number of words of this dimension to the total number of words. Among the 102 dimensions, five that are closely related to emotions were selected to measure emotions expressed in postings, which are: “positive emotion”, “negative emotion”, “anxiety”, “anger”, and “sadness”. The independent samples *t*-test was employed to test whether there were significant differences between Depression Super Topic and the regular timelines in these five dimensions.

In social media, users can like and comment on each other’s posts to deliver informational support or emotional support. To explore whether people with depression could receive more support in this online community, we compared the number of likes and comments between Depression Super Topic and regular timelines. The 5000 selected users were used as the sample. After calculating the mean number of likes and comments for each of the 5000 selected users, the Wilcoxon signed-rank test was used to measure the difference of the number of likes and comments between Depression Super Topic and the regular timelines [26].

To study the diurnal activity patterns of people with depression, we analyzed the posting time of messages from Depression Super Topic and regular timelines. The percentage of messages posted per hour was calculated to measure the diurnal patterns. In comparison analysis, each day was divided into two periods: day and night. We calculated the number of postings in Depression Super Topic and the regular timelines for each period. Pearson chi-square test was then employed to test whether there is a significant difference in posting time between the two.

### 2.4. Statistical Analysis

The independent samples *t*-test was conducted by SPSS version 19.0 (IBM, Armonk, NY, USA). The Wilcoxon signed-rank test and Pearson chi-square test were carried out with the assistance of the SciPy version 1.7.0 in Python [27].

## 3. Results

### 3.1. Themes of Distilled Posts

From the 239 crawled distilled posts, themes were identified to learn the needs and interests of members in Depression Super Topic and expected moderation of this online community.

Table 1 displays the five themes found in distilled posts. Postings about regulations on community management and warnings on inappropriate posts suggest there are strict regulations within Depression Super Topic. Management is conducted through the statement of the regulations and public accusations of undesirable behavior, such as uploading content about self-harm or suicide, throwing insults, defrauding, and selling counterfeit medicines. Postings about emotional support contained self-support and support for other people with depression. The information shared in informational support indicates members’ information needs, such as scheduling an appointment with a psychologist, introducing antidepressants, need for hospitalization, and recommendations of psychiatrists and hospitals. Emotional disclosure and life sharing accounted for the same proportion as informational support, among which positive emotions and negative emotions are both conveyed. The rest of the postings initiated discussions that encouraged members to communicate in the comments section.

### 3.2. Comparison

Table 2 and Table 3 provide overviews of the topics in regular timelines and Depression Super Topic, respectively. In the regular timelines, only 51.9% of the postings mentioned personal life, emotions, and thoughts. The remaining postings were about news and supporting favorite celebrities in activities initiated by Sina Weibo. In Depression Super Topic postings, expressing emotions was the most common topic. Nearly one-fourth of the postings were about philosophical thoughts of life; 13.1% of the postings were about emotional support; and 12.6% of the postings mentioned personal life. Treatment of depression, including sharing treatment experiences, doubts about diagnosis, and questions on antidepressants, accounted for more than 10% of the postings, closely followed by physical effects of depression.

**Figure 2:**
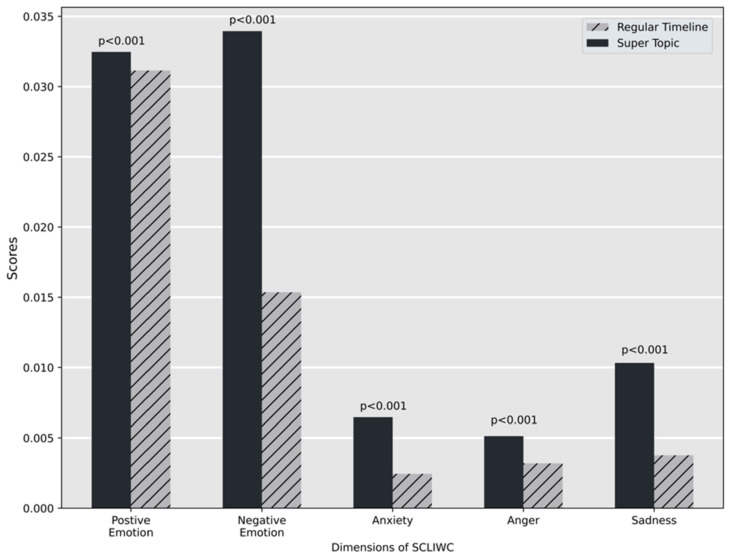
SCLIWC scores for selected dimensions.

The independent samples *t*-test suggests that the difference of emotions expressed is significant across all five dimensions (*p* < 0.001). Figure 2 provides an overview of the mean scores for each dimension. Our analysis showed that posts published in Super Topic expressed more emotions than the regular timelines.

**Figure 3:**
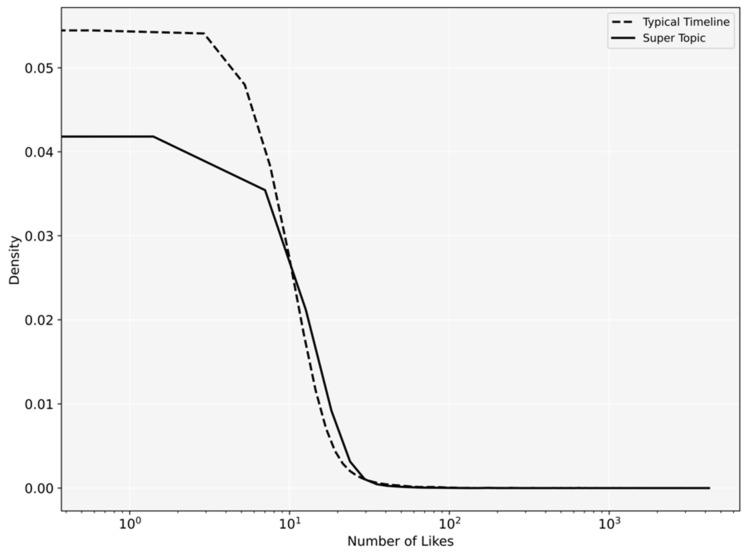
Probability density of likes users received in Depression Super Topic and regular timelines.

**Figure 4:**
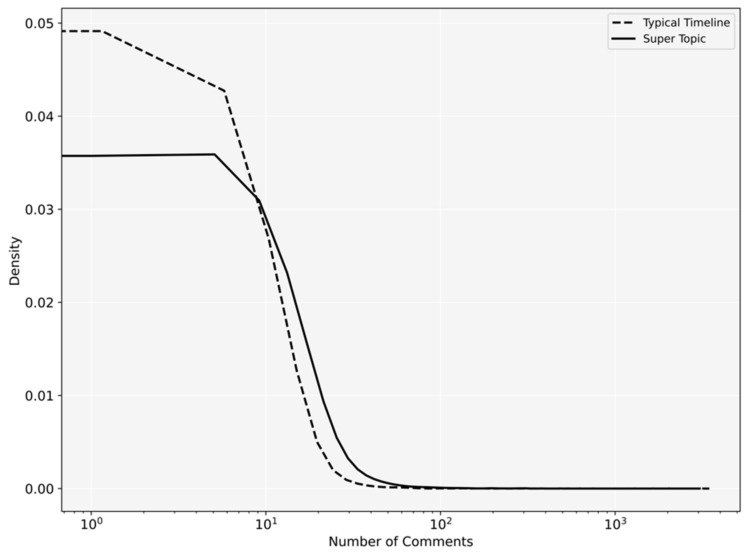
Probability density of comments users received in Depression Super Topic and regular timelines.

Figure 3 and Figure 4, respectively, show the probability density of the number of likes and comments. The posts published in Depression Super Topic received significantly more comments (*p* < 0.001) and more likes (*p* = 0.009) compared to the posts published in regular timelines.

**Figure 5:**
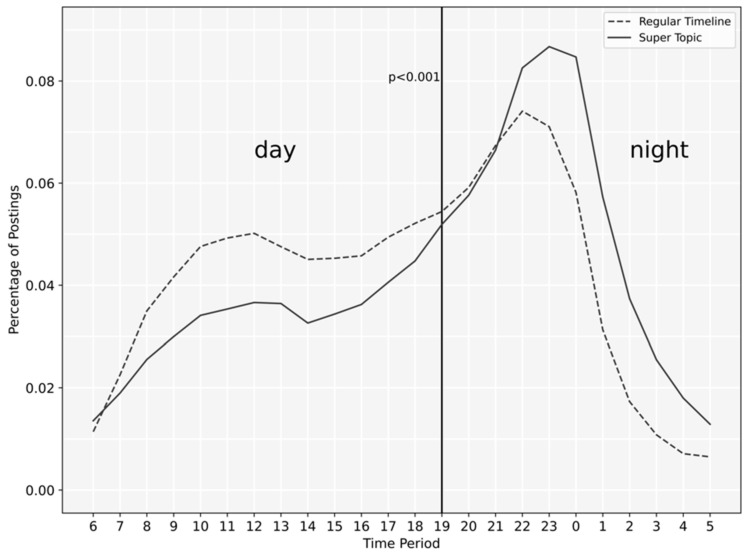
Diurnal activity patterns of users in Depression Super Topic and regular timeline.

Figure 5 shows the proportions of postings for a 24-h day. Pearson chi-square suggests that the diurnal activity patterns between Depression Super Topic and the regular timeline are statistically significant (*p* < 0.001), showing that people with depression tended to be more active in Depression Super Topic during sleeping hours (i.e., 21:00–06:00).

## 4. Discussion

This paper examined what happens when people with depression gather online by identifying the themes of distilled posts and comparing members’ postings published in the community and their regular timelines. This comparison helps to analyze the impact of the gathering of people with depression online. The distilled posts reflect self-moderation within this online community, which indicates the content to pay attention to and the behaviors that should be banned.

From studying the content of distilled posts, the most common theme that emerged was regulations on community management and warnings of undesirable content, closely followed by emotional support, informational support, as well as emotional disclosure. The posts about regulations suggest that this online community is administrated, and the moderators continuously guide and shape the community into a positive and healthy platform. But the content of warnings also indicated that some members were about to commit suicide, and some were at risk of getting hurt by other members. A more stringent supervision system should be set in this community to effectively react to inappropriate behaviors in a timely manner. The posts about “emotional support” and “informational support” represented the support needs of depressed individuals that could not be found elsewhere. Because this community is almost totally formed of people with depression, most support is delivered by individuals with real personal experience. Prior research has shown that peer support from other patients is effective for curing depression [28]; seeking information from other people’s experiences provides understanding, hope, and empathy [29]. Therefore, in terms of gaining support, an online depression community is beneficial for people with depression. Murphy found that the disclosure of similar experiences could increase the feeling of friendliness and commonality [30]. Therefore, Depression Super Topic postings about similar experiences and feelings may enhance empathy and hope for members. This may explain why postings on emotion disclosure and life sharing received replies and were selected as distilled posts. Additionally, being selected as distilled can be considered a socio-affective response, which is also beneficial to the posters [31].

In Depression Super Topic, the identified topics cover members’ personal life and thoughts, physical effects brought by depression, treatment of depression, and support for peers, all of which are related to depression. It is noteworthy that in the regular timeline, more than one-third of the postings are about supporting favorite celebrities. One possibility is that admired celebrities could instill a sense of hope for people with depression. What is more, joining a fan club is also a means of seeking a sense of belonging, similar to following an online depression community.

A significant difference in both negative and positive emotions between Depression Super Topic and regular timelines was observed. It suggests that Depression Super Topic provides a platform to vent emotions freely. However, prior research has shown that both positive emotions and depression can spread through social networks, and the contagious “influence” is cumulative with the number of contacts [32]. In this community, by exposing themselves to other people with depression’s complex emotions, members are at risk of being negatively influenced by them.

In Depression Super Topic, people with depression can receive both more comments and likes than on their regular timelines. Especially, the difference of comments is highly significant according to the statistical analysis. In social media, likes and comments are both activities to interact with others, but comments fall on a higher level than likes [33]. In Depression Super Topic, members could provide both information and emotional support to other people with depression through commenting, while likes could only indicate approval. More comments suggest that Depression Super Topic is meeting support needs and providing a platform to communicate.

The diurnal pattern analysis suggested that people with depression are more active during the night, which concurs with previous findings [34,35]. Our study further showed that they prefer to post in the depression online community during sleeping hours, which is not surprising since insomnia is a common complaint of people with depression that in turn triggers loneliness [36,37]. Posting messages in Depression Super Topic enables people with depression to vent internal emotions and communicate with peers, thereby ameliorating their loneliness.

It should be noted that there were some limitations to our study. Firstly, there are no verification processes in the subscription procedures, so we cannot guarantee that all the members are people with depression. Secondly, only 203,659 postings were crawled from Depression Super Topic, which was a relatively small proportion of all the posts. Finally, since we only studied one community in Sina Weibo, the findings may not be generalized.

## 5. Conclusions

This paper explored what happens when people with depression gather online by analyzing the postings from Sina Weibo’s Depression Super Topic and comparing them with regular timeline postings. Moderators administrate the Depression Super Topic online community to make it a positive platform for people with depression to receive emotional and informational peer support online, which is not otherwise available. Findings from the research suggest that people with depression are more likely to disclose their thoughts and feelings in an online depression community than on their regular timelines and that they tend to be more active during the night. This study contributes to an understanding of the needs of those suffering from depression and can act as a guide to targeted support for them.

## Figures and Tables

**Table 1:** Themes of distilled posts and sample posts.

Theme	*n* (%)	Example Posts
Emotional support	57 (23.8%)	I have beaten the big black dog! I wish all of you could recover soon.
Informational support	55 (22.9%)	I’ve had two psychological consultations, wish this article could be helpful for you.
Regulations on community management and warnings of undesirable content	58 (24.2%)	Prohibiting posting messages about suicide and selfharm. Prohibiting posting messages about medicine dealing.
Emotion disclosure and life sharing	55 (22.9%)	Want to share something beautiful with you.
Initiating discussions	14 (5.8%)	How do you think that health insurance should cover medicines for depression?

**Table 2:** Themes of postings from regular timelines.

Label	*n* (%)	Top 30 Keywords	Example Postings
Support Celebrities	75,487 (37.7%)	Super Topic, together, ranking list, hurry, hahaha, Zhan Xiao, red package, come on, weibo, star, click, panda, influential power, get, join, still, protector, up, boost popularity, fans, vote, already, zone, popularity, most, music, contribute, idol original image, a group of images, total, Super Topic, display, map, share, little, Yilong Zhu, lonely, eat, today, image, love, wallpaper, good, profile picture, wuwu(voice of cry), cute, happy, happy birthday, hottie, good-looking, like, Chenyu Hua, good night, Yishan Zhang, very, Xukun Cai	@Yibo Wang, I cheer up for you on the ranking list of celebrities in the mainland. You are my only life in this world. You are so great!
Personal Life and Feelings	103,704 (51.9%)	All, not, people, good, say, want, will, very, without, really, still, go, see, original image, make, know, up, is not, like, now, Super Topic, think, today, again, no, wish, love, inside, life	Suddenly I am feeling anxious and unpeaceful. The first business in this year! Pretty happy.
News	20,809 (10.4%)	Video, weibo, ahahah, China, shoot, second, day, share, month, inside, year, Communist Youth League, Roseanne Park, news, pandemic, after, red package, Qinghai, CCTV, America, new, card, covid-19 case, country, work, already, Wuhan, surprise, repost	British media claim that China’s concealment of the epidemic has delayed the prevention and control of the British outbreak, the Embassy in the UK response.

1. Since yixia and qilai have no practical meaning in Chinese characters, and there is no equivalent meaning in English, pinyin has been used. 2. As some words in Chinese match with one word in English, the number of keywords for one topic may not be 30. 3. For the topic “Support Celebrities”, integrated by two topics returned by LDA, two groups of keywords are both displayed in the table.

**Table 3:** Themes of postings from depression Super Topic.

Label	*n* (%)	Top 30 Keywords	Example Postings
Treatment	23,471 (11.5%)	Take/eat, doctor, take medicine, depression, medicine, everyone, hospital, is there any, go, yixia, want, good night, would/will, treatment, ask, read/see, anxiety, serious, major, feel, today, side effect, examine, diagnose, month, situation, do, recently	13 April. it is the first day of being diagnosed. Prescribed Sertraline, Wuling Capsules, Diazepam I have decided to go to the hospital. Are there any hospitals or gentle doctors in Shenzhen? What is the estimated cost?
Expressing emotions	59,234 (29.1%)	Good/better, want, really, not/no, all, very, unwilling, knows, feel, die, would/will, terrible, cry, think, without, every day, now, sad, tired, happy, do, emotion, qilai, seem, fear, sorry, a little, already, breakdown, still	My emotions are out of control tonight. I cried for a long time and afraid to make any noises. The swollen eyelids are heavy. I have been working hard and want to let others see the positive and optimistic me. But I am sad and anxious. I really hate the unhappy me and feel sorry for my family. They must be extremely disappointed in me. Can’t be happy anymore.
Emotional support	26,561 (13.1%)	Wish/hope, happy, live, world, without, die, life, all, good, cheer up, want, leave, step, again, one day, struggle, painful, inside, definitely, Weibo, everyone, don’t, video, meaning, future, one time, beautiful, love, but	I want to let it go and embrace life There are gifts, cakes, and red packets for my birthday, and I have received blessings from everyone. Thank you for your company and blessings! I hope everyone can live well, we support each other, complain, and tide over the difficulty of depression together!
Philosophical thoughts on life	49,489 (24.3%)	People, all, not/no, would/will, without, say, others, very, depression, think, isn’t, know, like, love, do, will, not, really, friend, maybe, many, things, actually, understand, someone, don’t, most, but, world	There is no such thing as empathy in the world. Others do not know how painful it is unless the needle pierces them. I will not say those comforting words, but I know that we must live. The most painful thing is not failure but is you could have done it.
Sharing life	25,681 (12.6%)	Say, go, not/no, still, without, want, all, today, see, know, mother, now, find, school, job, ask, friend, money, unwilling, teacher, on, tomorrow, scold, go home, home, parents, hospital, yixia	I was scolded as an orphan by my mother yesterday. I still feel hurt when I think about it. After taking medicine the first time, my mother asked: if you are a man? If you want to die, go to jump off the building, cut your wrists, and go on the rails.
Physical effects of depression	19,223 (9.4%)	Sleep, night, cannot sleep, all, today, still, without, on, insomnia, cry, hour, inside, feels, suddenly, fall asleep, morning, one day, now, lie, pain, dream, wake up, every day, a little, phone, noise/voice, would/will	When I returned from work in the past few days, I always had dizziness, headaches, difficulty breathing, almost panting, general weakness, trembling, and stiffness. I still have the same nightmare every night. It is really painful and uncomfortable. Now my memory is getting worse. I forgot what others said 10 min ago.

## Data Availability

The data are not publicly available as the data also forms part of an outgoing study.
